# Self-Cleaning Nanocomposite Membranes with Phosphorene-Based Pore Fillers for Water Treatment

**DOI:** 10.3390/membranes8030079

**Published:** 2018-09-07

**Authors:** Joyner Eke, Katherine Elder, Isabel C. Escobar

**Affiliations:** Department of Chemical and Materials Engineering, University of Kentucky, Lexington, KY 40506, USA; Joyner.Eke@uky.edu (J.E.); Katherine.Elder@uky.edu (K.E.)

**Keywords:** phosphorene, membranes, 2D materials, fouling, nanofiltration

## Abstract

Phosphorene is a two-dimensional material exfoliated from bulk phosphorus and it possesses a band gap. Specifically, relevant to the field of membrane science, the band gap of phosphorene provides it with potential photocatalytic properties, which could be explored in making reactive membranes that can self-clean. The goal of this study was to develop an innovative and robust membrane that is able to control and reverse fouling with minimal changes in membrane performance. To this end, for the first time, membranes have been embedded with phosphorene. Membrane modification was verified by the presence of phosphorus on membranes, along with changes in surface charge, average pore size, and hydrophobicity. After modification, phosphorene-modified membranes were used to filter methylene blue (MB) under intermittent ultraviolet light irradiation. Phosphorene-modified and unmodified membranes displayed similar rejection of MB; however, after reverse-flow filtration was performed to mimic pure water cleaning, the average recovered flux of phosphorene-modified membranes was four times higher than that of unmodified membranes. Furthermore, coverage of MB on phosphorene membranes after reverse-flow filtration was four times lower than that of unmodified membranes, which supports the hypothesis that phosphorene membranes operated under intermittent ultraviolet irradiation can become self-cleaning.

## 1. Introduction

Nanomaterials with tunable properties show promise for numerous technologies [1,2,3,4] because of their size-dependent electronic structure and controllable physical properties. Two-dimensional nanomaterials are materials that can be isolated as freestanding one atom thick sheets [5]. They are typically generated from bulk-layered crystalline solids [6]. These solids consist of successive layers of covalently bonded atomic layer planes ranging from one to multiple atoms thick, separated successively by van der Waals gaps [7]. Phosphorene distinguishes itself from other 2-D layered materials by its intrinsic structural anisotropic features [8]. Unlike graphene, phosphorene combines a high carrier mobility with a fundamental band gap [9], which imparts an intrinsic fine-tuning ability [10], thereby providing numerous opportunities for research. Specifically, relevant to the field of membrane science, the band gap of phosphorene provides it with electronic [11] and photocatalytic [12] properties, which could be explored in making reactive membranes that could simultaneously remove and destroy compounds. Using theoretical computational studies, Liang et al. [13] and Zhang et al. [14] studied the performance of self-passivated porous phosphorene membrane in hydrogen purification. The results showed excellent permeance and significant selectivity for hydrogen over carbon dioxide, methane, and nitrogen, which suggests that phosphorene shows potential for hydrogen purification. However, no experimental studies were performed.

Nanocomposite membranes are membranes that consist of polymeric or ceramic materials and nanomaterials. Nanoparticles can be deposited on the surface or embedded within the membrane matrix to impart useful functionality, enhance membrane separation, and anti-fouling properties [15]. Phosphorene exhibits a strong interaction with light, which is considered highly desirable in photocatalysis applications. With the high toxicity and corrosive issues encountered with metal-based photocatalysts (oxides, sulfides, and nitrides of titanium, tungsten, cadmium, and transition-metal dichalcogenides), phosphorene can act as a metal-free photocatalyst to degrade organic compounds in the feed solution to make reactive and self-cleaning membranes. Through liquid and/or mechanical exfoliation or direct synthesis, two-dimensional materials can be either assembled as a thin active layer on the membrane surface or incorporated into the membrane polymer matrix [16]. The degradation of phosphorene obtained by liquid-phase exfoliation occurs more slowly than that for phosphorene prepared by mechanical cleavage [17]; therefore, liquid-phase exfoliation of black phosphorus was chosen and was carried out in a basic medium, since this technique produces phosphorene with high water stability and controllable size and layer number [18].

**Figure 1:**
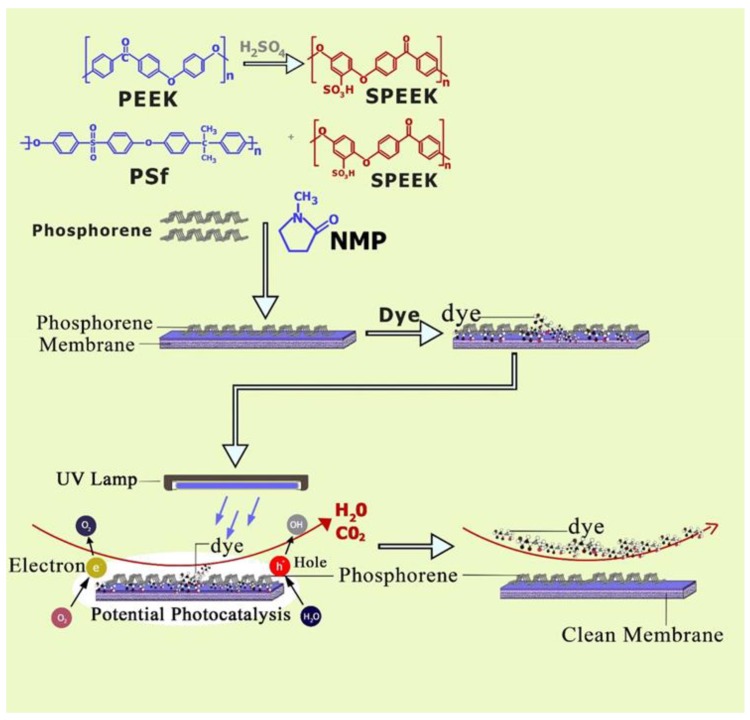
Preparation of a phosphorene-incorporated nanocomposite membrane.

The purpose of this study was, for the first time, to experimentally determine the viability of exfoliated phosphorene to be embedded in a polymer matrix in order to fabricate self-cleaning membranes. To fabricate membranes, a polymer blend was used to obtain a polymer material with properties intermediate between those of the pure components. The hydrophilic–hydrophobic balance, as well as other properties, such as physical structure and surface/pore charge of a membrane system, were altered since the membrane was prepared from a multi-component polymer blend [19]. For this study, the base membrane dope solution consisted of a blended polymer prepared by dissolving polysulfone (PSf) and sulfonated poly ether ether ketone (SPEEK) in a (95/5%) ratio into *N*-methyl pyrrolidone (NMP). SPEEK is a hydrophilic and negatively charged polymer with low permeability and mechanical strength; on the other hand, while PSf has good chemical resistance, high thermal stability, and good mechanical properties, it is hydrophobic and has poor solubility in solvents. The blend of PSf and SPEEK has been shown to result in a membrane with higher water permeability and permselectivity as compared to the pure polymers [20]. Using physical mixing between the blended membrane polymer dope and phosphorene, van der Waals interactions were formed between the constituents, and hence, phosphorene nanoparticles were incorporated into the dope solution. Methylene blue (MB) was filtered through the membranes under ultraviolet light, and the permeability and selectivity of the membranes were determined. The goal of this study was to determine if the addition of potentially photocatalytic phosphorene to polymeric membranes operated under intermittent UV irradiation would be able to produce self-cleaning membranes, as shown in Figure 1.

## 2. Experimental

### 2.1. Materials

Bulk black phosphorus was purchased from Smart Elements Inc., Vienna, Austria. Powdered PEEK, *N*-methyl-2-pyrrolidone (NMP), sodium hydroxide, and concentrated sulfuric acid (95–98%) were purchased from VWR, Radnor, PA, USA and Polysulfone (Solvay, Princeton, NJ, USA).

### 2.2. Exfoliation of Phosphorene from Black Phosphorus

The liquid exfoliation of black phosphorous (BP) was carried out using previously developed methods [18]. Bulk black phosphorus (15 mg) was added to 15 mL of NaOH and 15 mL of NMP solution in a ratio of 1:1. To exfoliate bulk black phosphorus, the mixture was sonicated using an ultrasonicator (P70H, Elma Elmasonic P, Singen, Germany) operated at 37 kHz frequency and 80% power for 4 h. After exfoliation, the solution was centrifuged at 3000 rpm for 10 min and it separated into two phases (exfoliated and non-exfoliated bulk BP), the non-exfoliated bulk BP was then discarded. The supernatant (exfoliated BP) was centrifuged at 4000 rpm for another 20 min to obtain fewer layers of phosphorene from NMP. The precipitations obtained were redispersed in water and the solutions were rinsed in deionized water for Raman studies. Raman studies were done on a silicon chip.

### 2.3. Membrane Preparation

The blended polymer dope solution was prepared by dissolving PSf and SPEEK (95/5%) into NMP. Exfoliated black phosphorus was added to the solution (0.5% wt/vol) and sealed with parafilm to prevent air bubbles from being trapped inside the solution and affecting the homogeneous mixing of the solvent and the solute. The blended solution was placed on a magnetic stirrer and heated at 65 °C. It was degassed in a sonicator to remove air bubbles for 1 h. The blended solution was spread on a glass plate with a doctor blade at a wet thickness of 0.250 mm and exposed to air for 12 s. A clean glass mirror was used as a surface, which provided optimum hydrophobicity to the membranes and helped the detachment of polymer films during phase inversion [21]. The glass plate and dope solution were immersed in a coagulation bath of deionized water and the membrane was formed via the process of phase inversion. The membranes formed were subsequently washed thoroughly with deionized water to remove residual solvent and kept in deionized water before testing. The thickness of the membrane was maintained at approximately 150 microns. By physical mixing between the blended membrane polymer dope and phosphorene, phosphorene nanoparticles were incorporated into the dope solution. Polymers that have similar solubility parameters with solvents are miscible [22], and closer values typically indicate better compatibility [23]. The solubility parameters of the polymers used for this experiment were the following: polysulfone, 21.2 (MPa^1/2^) [1] and sulfonated polyetheretherketone, 26.2 (MPa^1/2^) [24] and the solvent, NMP has a solubility parameter of 22.4 (MPa^1/2^) [25]. These values indicate that the membrane made with these polymers–solvent combinations should be stable.

### 2.4. Flux Analysis 

Flux analysis was performed in accordance with previously published studies [26] and will be summarized here. Filtration experiments were performed in batch mode but under continuous stirring using an Amicon filtration cell (Amicon Stirred Cell 8010–50 mL, Burlington, MA, USA). The method used to monitor the flux performance of the membrane was dead-end filtration. To determine flux through the membrane, the time to collect a 2-mL permeate sample was measured for each feed. Surface area and pressure were kept constant for the duration of the experiment. The active filtration area was 13.4 cm^2^ and the pressure was 2.06 bar (30 psi). Flux values were calculated as L/m^2^h and plotted against the total time of filtration. Membrane samples were supported with Whatman^TM^ filter paper (110 mm). Each membrane was precompacted with deionized (DI) water until a stable flux was reached. Precompaction was followed by filtration of dye solutions of 10 ppm each of methylene blue (MB) in water. The concentrations of the dye were determined using a bio-plate reader and the dye rejection was calculated according to Equation (1) [26]:

R = (1 − (C_p_/C_f_)) × 100% (1)

where C_p_ and C_f_ are solute concentrations in permeate and feed solutions, respectively.

After water filtration, reverse-flow filtration using DI water was performed to remove reversibly attached foulants that were not adsorbed to the membrane, and the filter paper support was changed. The flux recovery of the membrane was measured afterwards.

### 2.5. Filtration Experimental Setup

Phosphorene was immobilized into PSf-SPEEK membranes. The resulting membrane was tested for the photo degradation and mineralization of an organic dye, methylene blue (MB), under near-UV/Vis (Spectroline Model EA-160, Westbury, NY, USA) and in continuous operation mode. To examine the effects under visible light, two similar experiments were set up with the filtration cell, one completely covered by aluminum foil to prevent penetration of sunlight, and irradiated with UV light for 30 min, while the other was uncovered. The wavelength of UV was 365 nm. The permeates were analyzed via a bio-plate reader at 662 nm (Biotek Instruments, Winooski, VT, USA).

### 2.6. Contact Angle Measurement

Contact angle is a measure of the wettability of a surface. Here, a drop shape analyzer (Kruss DSA100, Matthews, NC, USA) was employed to carry out the contact angle measurement of all the membrane samples. The process for taking a measurement involved adding a small drop of water on the membrane surface and measuring the resultant angle of the droplet to the surface [27]. Hydrophilic materials display lower contact angles as compared to more hydrophobic materials.

### 2.7. Zeta Potential

Zeta (ζ) potential is the potential difference between the dispersion medium and the stationary layer of fluid attached to the dispersed particle [28]. It is used to determine the surface charge of materials under different pH environments. For this study, an Anton Paar SurPASS electrokinetic analyzer (Anton Paar, SurPASS, Ashland, VA, USA) in surface analysis mode was used. To ensure the removal of solvents from the membrane surface, membranes were rinsed with DI water before running analysis. The ionic strength of the potassium chloride electrolyte solution used in these measurements was 1.0 mM. Measurements were done under several pH environments and the pH was adjusted using 0.5 M NaOH and 0.5 M HCl solutions.

### 2.8. Raman Studies

The theory of Raman spectroscopy is discussed elsewhere [29,30], and briefly summarized here from those. When light is scattered from a molecule or crystal, most photons are elastically scattered. The scattered photons have the same energy (frequency) and, therefore, wavelength, as the incident photons. However, a small fraction of light is scattered at optical frequencies different from, and usually lower than, the frequency of the incident photons. The process leading to this inelastic scatter is termed the Raman effect. Raman scattering can occur with a change in vibrational, rotational, or electronic energy of a molecule. Raman scattering occurs only when the molecule is polarizable. If the scattering is elastic, the process is called Rayleigh scattering. If it is not elastic, the process is called Raman scattering [29,30]. Raman spectroscopy is a vibrational technique. In an inelastic process, like the Raman scattering, light is scattered and a phonon or normal mode is created or destroyed [31]. For a vibration to be active in a Raman spectrum, the vibration must change the polarizability of the molecule. Using the group theory and character tables, vibrational modes can be assigned to a molecule. Raman spectroscopy has been widely used to understand the electronic and vibrational properties, as well as their dependence on the thickness of various 2-D layered materials [32,33]. The pump radiation was supplied by a laser operating at a wavelength of 632 nm; the Raman emission was collected by a 100× objective in a backscattering geometry. A He-Ne laser was used at a power of 20 mW, but a neutral density filter was done on the sample so the laser spot on the sample had less than 0.1 mW.

### 2.9. Liquid–Liquid Porometer Studies

A porometer model LLP-11000A (PMI, Ithaca, NY, USA) was used in this study. The procedure involves a pair of immiscible liquids, of which the liquids used to wet the membrane, is referred to as the wetting liquid (isopropanol in this case), while the second liquid (sliwich oil) is used to displace it. By measuring the pressure and the flow through the membrane, the corresponding pore radius can be calculated using the Cantor Equation (2) [34] and the contact angle is assumed to be zero:

P = (2γcosɵ/r_p_) (2)

where P is the pressure, γ is the interfacial tension, θ is the contact angle, and r_p_ is the pore radius.

### 2.10. Morphological Characterization

A FEI Quanta 250 FEG Dual Beam Electron Microscope (FEI, Hillsboro, OR, USA), which has an energy dispersive X-ray spectroscope (EDX) attached to it (Oxford Instruments, X-Max), was used to characterize the samples here. To visualize clearer images, small samples of the membranes were frozen in liquid nitrogen before cutting. Cross-section imaging of the membranes was achieved by vertical attachment to a carbon tape, while surface imaging of the samples was achieved by horizontal attachment. The surfaces of the samples were sputtered with a thin layer of palladium-gold using a sputtering device (Emscope SC400, Kent, UK) and then observed under a scanning electron microscope, and then the EDX analysis was performed on the sample.

### 2.11. Surface Fluorescence Characterization

To determine the level of fouling by MB after each experiment, the membranes were imaged under a fluorescent microscope. Images where recorded on a Zeiss 880 NLO upright confocal microscope (Thornwood, NY, USA) with a 10× air objective. The membranes were sandwiched between microscope slides and wetted with water for smoothing them out. Tiles, showing the full field of over a *z*-range to cover the slightly non-planar geometry of the membrane, were stitched together in a format of 3 × 3, reflecting a representative cover. Methylene blue was excited with a 633 nm laser and emission was collected over a spectral range of 642 to 759 nm.

## 3. Results and Discussion

**Figure 2:**
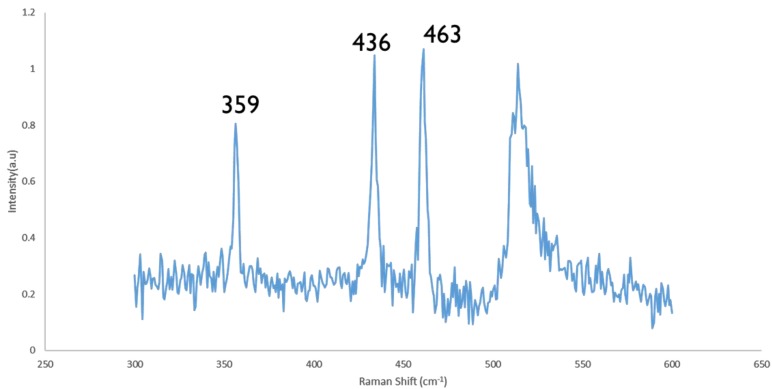
Phosphorene characteristic Raman bands at 359 cm^−1^, 436 cm^−1^, and 463 cm^−1^.

Using dynamic light scattering, the average hydrodynamic diameter of the phosphorene nanoparticles after exfoliation was found to average 1.87 nm. To confirm that few-layer phosphorene was fabricated, thin phosphorene films were first identified using optical microscopy before being studied under the Raman microscope. Raman spectroscopy was used to analyze few-layer phosphorene (i.e., between 2–5 layers) after exfoliation. Sample analysis was performed under ambient conditions. As seen in Figure 2, Raman bands were observed at 463 cm^−1^, 436 cm^−1^, and 359 cm^−1^, assigned to the one out-of-plane mode A^1^_g_ and two in-plane modes, B_2g_ and A^2^_g_ (A^1^_g_, B_2g_, and A^2^_g_ represent vibrational modes) of few-layer phosphorene corresponding to observed values from the literature [35].

**Figure 3:**
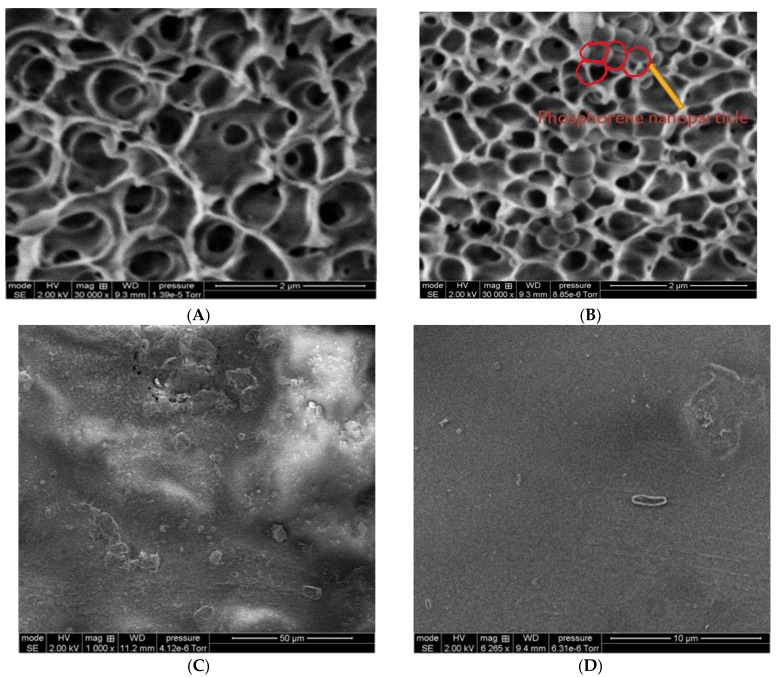
Cross-section SEM analyses of (**A**) SPEEK membranes and (**B**) phosphorene-membranes with some of the phosphorene nanoparticles marked in red, and surface SEM analyses of (**C**) SPEEK membranes (50-micron magnification) and (**D**) phosphorene-membranes (10-micron magnification) before filtration.

Figure 3A,B shows the cross-section of the pore structure of SPEEK membranes before and after the addition of phosphorene, respectively, while Figure 3C,D shows the surface images of both membranes before filtration. By comparing the two images, it was confirmed that phosphorene was immobilized onto the membranes. The phosphorene membranes showed spherical-looking structures present in the pores, which upon analysis by EDX, were confirmed to come from phosphorus. Phosphorene nanoparticles were blended with the dope solution before casting the membrane, and while care was taken to prevent agglomeration, nanoparticle agglomeration still occurred, and it is believed that the increase in nanoparticle size after casting was likely due to agglomeration.

**Figure 4:**
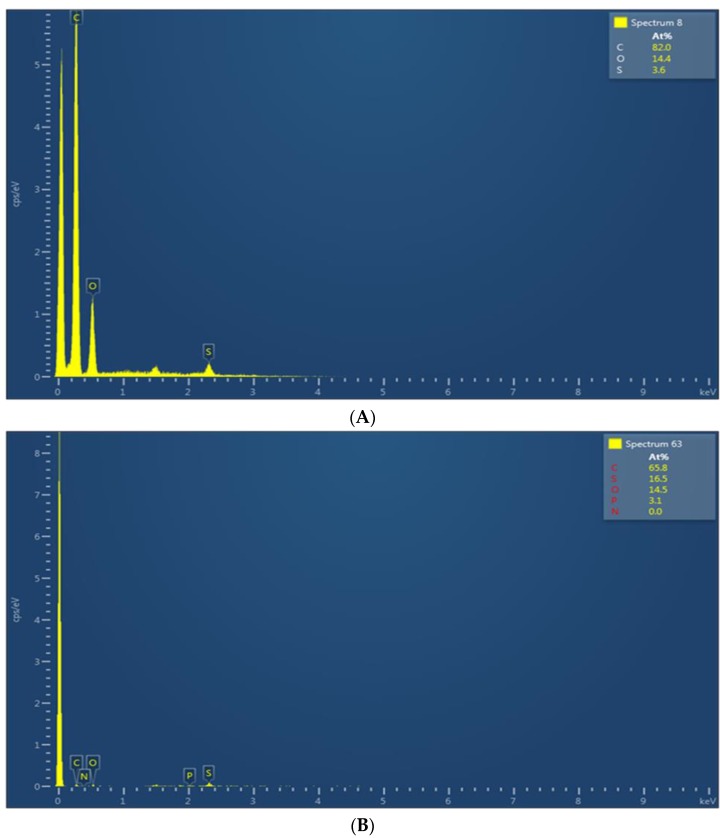
(**A**) EDX spectrum of SPEEK membrane showing the presence of carbon, oxygen, and sulfur. (**B**) EDX spectrum of phosphorene containing SPEEK membrane showing the presence of carbon, oxygen, sulfur, and phosphorus.

Figure 4A,B show the associated EDX spectra for the SPEEK and phosphorene membranes, respectively. From Figure 4A, SPEEK membranes contained 82% carbon, 14.4% oxygen, 3.6% sulfur, and no detectible phosphorus. On the other hand, the EDX spectrum of the membranes incorporated with phosphorene (Figure 4B) show 65.8% carbon, 14.5% oxygen, 16.5% sulfur, and 3.1% phosphorus. Therefore, both Raman and EDX analyses support the exfoliation of few-layer phosphorene and the subsequent the presence of phosphorene on the membranes, respectively. Since SPEEK has no phosphorus, all the phosphorus fraction measured was due to the presence of phosphorene on the membranes, and it amounted to 3.1% phosphorus. 

The pore diameter at the maximum pore distribution, i.e., the most prevalent pore size, of the SPEEK membranes was on average 0.022 microns (with smallest and largest detected pores being 0.017 and 0.086 microns), while that of the phosphorene membranes averaged 0.0024 microns (with smallest and largest detected pores being 0.0022 and 0.0078 microns), which further indicates the addition of phosphorene accumulating within the pores, in agreement with Figure 3B, and put the membranes in the nanofiltration range. Phosphorene membranes also displayed different pore size distributions, with pore sizes not being as uniform when compared to the baseline SPEEK membranes, hence the high standard deviation. This again showed good agreement with scanning electron microscopy (SEM) images (Figure 3) since Figure 3B shows that phosphorene accumulated in some of the pores of the membranes, which would lead to the formation of smaller, non-uniformly distributed pores.

**Figure 5:**
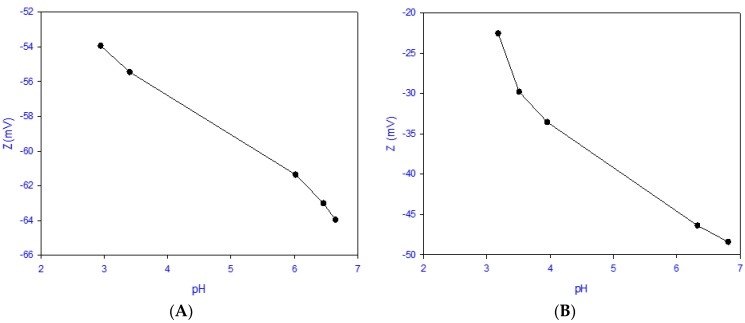
Surface charge vs pH plot of (**A**) SPEEK membrane and (**B**) phosphorene-modified membrane.

SPEEK membranes displayed an average hydrophilicity as measured by contact angle of 48.3° ± 0.67°, while phosphorene-membranes had an average contact angle of 81.5° ± 0.64°. This shows that unmodified membranes were more hydrophilic, while phosphorene membranes had a more hydrophobic nature that is associated with the presence of the more hydrophobic phosphorene [36]. The switch from a more hydrophilic to a more hydrophobic membrane further supports that the chemistry of the membrane had changed, which was due to the addition of phosphorene. To further characterize changes incurred by the addition of phosphorene, the surface charge was evaluated, as shown in Figure 5. It was observed that both SPEEK membranes and phosphorene membranes were negatively charged in both acidic and basic mediums. At a pH of approximately 6, the zeta potential of SPEEK was −61 ± 4.6 mV while that of the phosphorene membrane was −44 ± 7 mV, which was possibly due to the phosphorene nanoparticles masking some of the sulfonic sites (the source of the negative charge of the membranes).

**Figure 6:**
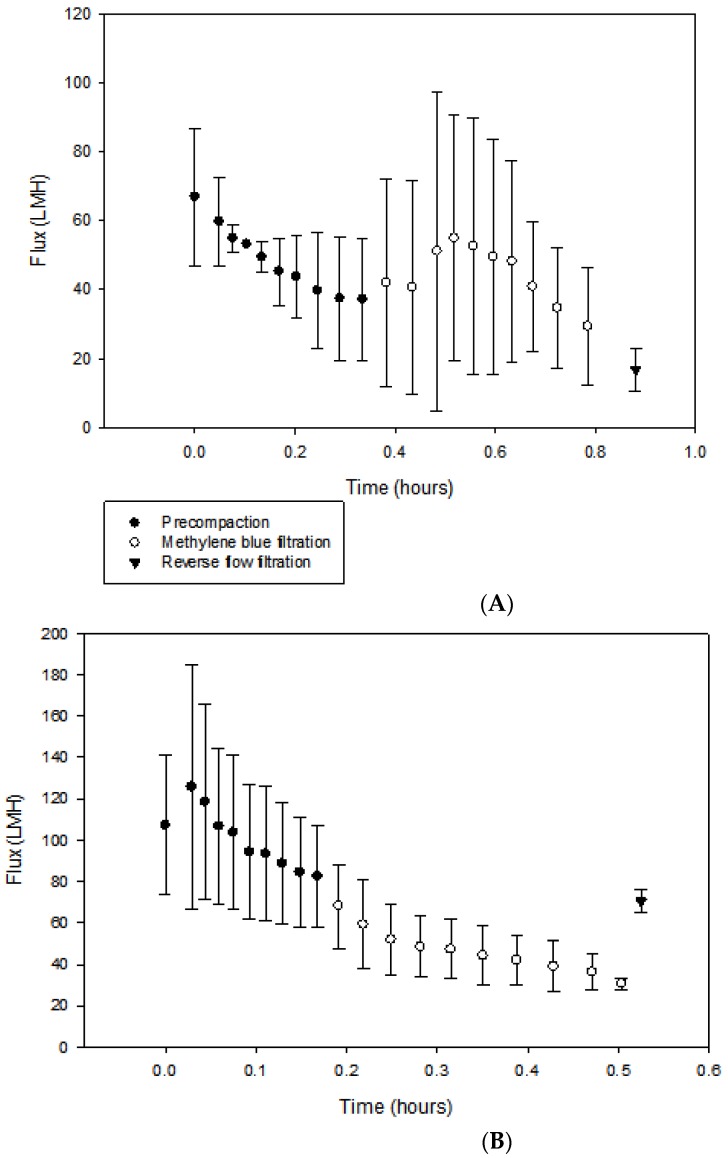
(**A**) Flux analyses of the SPEEK membrane under UV irradiation at a constant pressure of 30 psi (2.06 bar). (**B**) Flux analysis of the phosphorene-modified membrane under UV irradiation at a constant pressure of 30 psi (2.06 bar).

By employing dead-end filtration, flux studies were carried out on the membranes under intermittent UV light irradiation. Precompaction, or filtration of pure water, was first performed to ensure that all solvents used during the membrane fabrication process were removed from the membranes’ surfaces and pores. As seen in Figure 6A,B, the average initial pure water flux values for SPEEK and phosphorene membranes were 67 ± 20.0 LMH and 107 ± 33.6 LMH, and the flux values at the end of precompaction were 37 ± 17.8 and 82 ± 24.9 LMH, respectively. Reasons for the high standard deviation include the fact that membrane samples were fabricated in laboratory-scale batch processes, and reaction completion was determined via reaction time; therefore, each batch could have slight differences when compared to others. Furthermore, small pieces of membrane were cut out for each experiment, having an area of 13.4 cm^2^. Both membranes obtained MB rejections of approximately 89%. While average values were different, standard deviations show that flux values of SPEEK and phosphorene membranes were not significantly different from each other. The likely reason for the higher flux values might have been because phosphorene membranes were more sponge-like, and hence more porous, as compared to SPEEK membranes, which was evident from the SEM images (Figure 3).

After precompaction was completed, MB solutions were filtered through the membranes, and after filtration, membrane cleaning was simulated via reverse-flow filtration using pure water in order to investigate the potential for cleaning. Initial MB solution filtration displayed flux values of 42 ± 30.1 and 68 ± 20.3 LMH for SPEEK and phosphorene membranes, respectively, while final flux values were 29 ± 16.9 and 30 ± 2.7 LMH for SPEEK and phosphorene membranes, respectively. The decrease in flux values during filtration showed that for both SPEEK and phosphorene, MB accumulated on the surface of the membranes to foul them. To measure the ability of phosphorene’s photocatalytic properties in self-cleaning the membranes under UV irradiation, the recovered fluxes were monitored. It was determined that for the SPEEK and phosphorene membranes, the recovered flux values were 17 ± 6.3 (or 45% of the initial pure water flux) and 70 ± 5.8 LMH (or 85% of the pure water initial flux, or a flux value similar to that at the start of MB filtration). Only after UV irradiation, the flux of phosphorene membranes was significantly higher and different as compared to SPEEK membranes.

Table 1 summarizes the flux values obtained. It was hypothesized that the membranes could become self-cleaning under the intermittent application of UV irradiation. This was verified by performing experiments using phosphorene membranes operated with and without UV irradiation. Under visible light (i.e., without UV irradiation), the recovered flux after reverse-flow filtration with pure water using phosphorene membranes was 35% of the initial flux at the start of MB filtration (i.e., the initial flux was 71 LMH and the recovered flux was 25 LMH). On the other hand, the phosphorene membrane operated under UV irradiation showed a full recovery of flux after reverse-flow filtration using pure water (i.e., the initial flux was 68 LMH and the recovered flux was 70 LMH). With the only variable being the presence of UV irradiation, and both membranes showing similar MB rejection at approximately 89%, it is hypothesized that the MB accumulated on the surface of the membrane was potentially destroyed, which would make the membrane self-cleaning.

**Figure 7:**
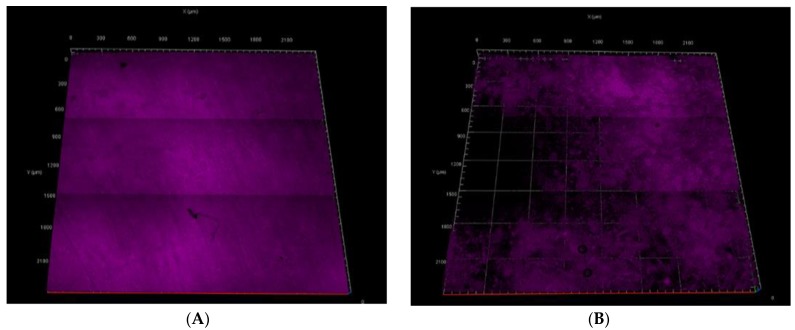
(**A**) Fluorescent image of SPEEK membrane after the reverse-flow filtration. (**B**) Fluorescent image of phosphorene-modified SPEEK membrane after the reverse-flow filtration.

Figure 7A,B show images of the MB stained membranes to evaluate the amount of MB that accumulated after reverse-flow pure water filtration, which provides a qualitative measure of the amount of MB that remained intact and irreversibly attached to the membrane. SPEEK membranes showed full coverage of MB, while phosphorene membranes did not show a uniform coverage of methylene blue under fluorescence. The SPEEK membranes had a coverage four times higher than that observed with the phosphorene membranes. This decrease was possibly due to the destruction of MB, and it agreed with the higher flux recovery values obtained when using phosphorene membranes. It is proposed that because phosphorene has a band gap that can be tuned sufficiently for photon absorption in the ultraviolet region, photocatalysis of the dye may have occurred under UV irradiation. This made the phosphorene membranes self-cleaning, as observed by a significantly higher flux recovery.

## 4. Conclusions

For the first time, nanocomposite membranes were fabricated using phosphorene. This opens the field to a new class of potentially reactive membranes, or at the least, easier to clean membranes. Due to phosphorene’s properties, these membranes have the potential to be used for multiple purposes, such as compound destruction, self-cleaning, biofilm formation prevention, etc. Membrane separations of the future will not favor static membranes, i.e., membranes that only serve the function of rejecting compounds, since accumulated and potentially hazardous compounds on the surface will be released on backwash/cleaning water to make that hazardous and make the membranes hazardous at the time of disposal. Hence, dynamic self-cleaning membranes that can simultaneously remove compounds and destroy them provide the field with an alternative. There are numerous reactive membranes in existence, but phosphorene brings tunable properties that open up a new field for research.

## Figures and Tables

**Table 1:** Flux values of phosphorene membranes operated under UV irradiation and without UV irradiation.

	Phosphorene Membranes		Phosphorene Membranes	
Flux Type	Operated without UV		Operated with UV	
	Flux (LMH)	St. dev	Flux (LMH)	St. dev
PWF Initial	56	2.6	107	33.6
PWF Final	74	7.1	82	24.9
MB Initial	71	12.5	68.1	20.3
MB Final	57	10.8	31	2.7
Recovered	25	5.3	70	5.8
